# Blood Monocyte Subsets with Activation Markers in Relation with Macrophages in Non-Small Cell Lung Cancer

**DOI:** 10.3390/cancers12092513

**Published:** 2020-09-04

**Authors:** Iwona Kwiecień, Elżbieta Rutkowska, Małgorzata Polubiec-Kownacka, Agata Raniszewska, Piotr Rzepecki, Joanna Domagała-Kulawik

**Affiliations:** 1 Laboratory of Hematology and Flow Cytometry, Department of Internal Medicine and Hematology, Military Institute of Medicine, 04-141 Warsaw, Poland; erutkowska@wim.mil.pl; 2 Department of Surgery, Institute of Tuberculosis and Lung Diseases, 01-138 Warsaw, Poland; m.polubiec@igichp.edu.pl; 3 Department of Pathology, Medical University of Warsaw, 02-106 Warsaw, Poland; agataraniszewska@vp.pl; 4 Department of Internal Medicine and Hematology, Military Institute of Medicine, 04-141 Warsaw, Poland; przepecki@wim.mil.pl; 5 Pulmonary Diseases and Allergy, Department of Internal Medicine, Medical University of Warsaw, 02-097 Warsaw, Poland; jdomagala@wum.edu.pl

**Keywords:** monocytes, macrophages, bronchoalveolar lavage fluid, CD62L, lung cancer microenvironment

## Abstract

**Simple Summary:**

This study characterized monocyte subtypes: classical, intermediate, and non-classical with the expression of surfaces markers: CD62L, CD11c, CD18, HLA-DR in non-small cell lung cancer patients (NSCLC) compared to healthy controls and correlations between monocyte subtypes and macrophages in the lung cancer microenvironment. We confirmed the presence of various monocyte subtypes in the blood with predominance of classic monocytes and a higher proportion of classical and intermediate monocytes in NSCLC patients than in healthy subjects. Our observation that intermediate monocytes with CD11c+ and HLA-DR+ expression correlation with the amount of macrophages from the lung cancer microenvironment may indicate role of these cells in cancer immunity. A high proportion of monocytes with low expression of CD62L indicates participation of monocytes in attenuation of anticancer response. The detection and monitoring of the presented monocyte subsets in the blood might be a useful test in lung cancer.

**Abstract:**

(1) The cells from the monocyte line play an important role as regulators of cancer development and progression. Monocytes present pro- and anti-tumor immunity and differentiation into macrophages. Macrophages are predominant in the lung cancer environment and could be evaluated by bronchoalveolar lavage fluid (BALF). (2) The aim of the study was analysis of monocytes: classical, intermediate and non-classical with expression of: CD62L, CD11c, CD18, HLA-DR in non-small cell lung cancer (NSCLC) and their correlation with BALF macrophages from lungs with cancer (clBALF) and healthy lungs (hlBALF). (3) A total of 24 patients with NSCLC and 20 healthy donors were investigated. Monocyte subtyping and macrophage counts were performed by flow cytometry. (4) There are three types in peripheral blood (PB): classical monocytes (CD14++CD16-), intermediate (CD14+CD16+) and non-classical (CD14-/+CD16++). We noticed a higher proportion of classical and intermediate monocytes in lung cancer than in healthy donors (76.2 vs. 67.3, and 7.9 vs. 5.2 *p* < 0.05). We observed a higher proportion of macrophages in clBALF then in hlBALF. A higher CD62L expression on all monocyte subtypes in healthy donors than in study group was found. There were positive correlations between: classical CD11c+, intermediate CD11c+, intermediate HLA-DR+ monocytes in PB with macrophages in clBALF. We did not observe these correlations with macrophages from hlBALF. (5) A predominance of classical and intermediate monocytes in lung cancer and the correlation between intermediate monocytes with CD11c+ and HLA-DR+ and macrophages from the NSCLC milieu support a role of monocyte-line cells in cancer immunity. A high proportion of monocytes with low expression of CD62L indicates the participation of monocytes in attenuation of anticancer response.

## 1. Introduction

The cells from the monocyte line play a role in cancer biology. Recently, monocytes have emerged as important regulators of cancer development and progression, with different subsets appearing to have opposing roles in enabling tumor growth and preventing metastatic spread of cancerous cells [1]. Monocytes may present pro- and anti-tumor immunity, including secretion of mediators, stimulation of phagocytose, promotion of angiogenesis, recruitment of lymphocytes and differentiation into macrophages. Macrophages are predominant in the lung cancer environment and are important inflammatory cells that regulate innate and adaptive immunity in cancer. Macrophages can generally exert anti-tumor cytotoxicity [2]. However, subsequent studies demonstrated that tumor-associated macrophages (TAMs) which are presented at tumor sites have a potential capacity to exert immune suppressive activities in most cases [3]. Regarding the role of macrophage precursors—monocytes in the cancer environment, the data on this topic are not entirely clear. Previous studies looking at monocytes in a range of different cancer types have demonstrated conflicting results regarding monocyte phenotype and function. Studies in patients with lung, breast and other cancers have described hindered monocyte function [4,5], whereas another study suggested that non-small lung carcinoma (NSCLC) does not affect monocyte adherence and phagocytosis in lung cancer patients compared to healthy controls. Other studies demonstrated that monocytes are capable of both inhibiting and stimulating tumor growth [6].

Recently advanced immunology research has discovered that monocytes are heterogenic and can be divided into three subsets based on specific surface markers and that each subset displays specific functions: classical (~85%), intermediate (~5%), and non-classical (~10% of monocyte population), which are characterized by their level of cluster of differentiation CD14 and CD16 expression [7]. The expression pattern of these surface markers lends some insight into their function. CD14 acts as a co-receptor for toll-like receptor 4 and mediates lipopolysaccharide (LPS) signaling, while the CD16 antigen is identified as FcγRIIIa and participates in innate immunity [8]. The proportions of monocyte subsets can differ with the presence and state of disease [9].

In addition, monocytes may express other antigens on their surface that significantly affect their functions.

CD62L, also known as L-selectin, is a cell adhesion molecule that play important role in lymphocyte–endothelial cell interactions. CD62L is expressed on neutrophils, monocytes, T- and B-lymphocyte subsets and NK cells [10]. Following cell activation CD62L is widely shed off. The adhesion molecule CD11b, a member of the β2-integrin family, strengthens the initial contact induced by CD62L and is involved in the migration into the inflamed tissue [11]. CD62L play a role in regulating recruitment of monocytes to tissue from the blood during inflammation. No differentiated monocytes circulate in the blood and then are recruited to the tissues where they differentiate into macrophages or myeloid dendritic cells [12]. Subsets of blood monocytes with differential migratory potentials have been identified, and the capacity of monocytes to preferentially migrate to sites of inflammation has been linked to expression of the selectin CD62L [13].

CD11c is an adhesion glycoprotein found at a high level on most human dendritic cells, but also on monocytes and macrophages. It associates with CD18 to form the CD11c/CD18 complex that binds fibrinogen and has been reported to be a receptor for iC3b and intercellular adhesion molecule 1 (ICAM-1). CD11c/CD18 plays a role as an adhesion molecule that mediates cellular binding to ligands expressed on stimulated epithelium and endothelium [14].

CD18 is also known as Integrin beta chain-2. Integrins are integral cell-surface proteins composed of an alpha chain and a beta chain, crucial for cells to be able to efficiently bind to the extracellular matrix [15] especially in extravasation neutrophil from the blood vessels. Integrins are also very important molecules for monocytes, CD18 is expressed on lymphocytes, monocytes, and more weakly on granulocytes. The known binding partners of CD18 are CD11a, CD11b, CD11c and CD11d. Binding of CD18 and CD11 results in the formation of Lymphocyte Functions Associated Antigen 1 (LFA-1) [16], which plays an important role in cellular adhesion in immune and inflammatory responses. Deficiencies in CD18 expression can lead to adhesion defects in circulating cells in humans, reducing the immune system’s ability to fight off pathogens [17,18].

HLA-DR plays a vital role in the immune response by regulating the interaction between antigen-presenting cells including monocytes. It has been described as a marker in monocyte–macrophage system. Monocytes express HLA-DR molecules, which are responsible for showing antigens to T cells, thereby presenting antigens to adaptive immune system cells [19]. Cytokines released by monocytes mediate responses to other cells and activate complement and coagulation cascades [20].

The aim of this study was to characterize monocyte subtypes: classical, intermediate, and non-classical with the expression of surfaces markers: CD62L, CD11c, CD18, HLA-DR in NSCLC patients compared to healthy controls. The next step was to find a correlation between monocyte subtypes and macrophages in the lung cancer microenvironment. We investigated the proportion of macrophages in two compartments based on the examination of bronchoalveolar lavage fluid (BALF): from the lung affected by cancer (clBALF—local cancer environment) and from the healthy lung (hlBALF—an internal control) from each patient and we evaluated the relations between them and monocytes in peripheral blood (PB).

## 2. Results

The clinical characteristics of the investigated group are summarized in Table 1. Most patients in the study group were in a non-advanced stage of lung cancer (stage I). The highest incidence was for adenocarcinoma (*n* = 16). Due to a small number of patients in each group we did not perform a comparison between groups with different types of cancer and between different stages of the disease.

The proportion of monocytes and macrophages in the studied groups is presented in the Table 2.

**Figure 1:**
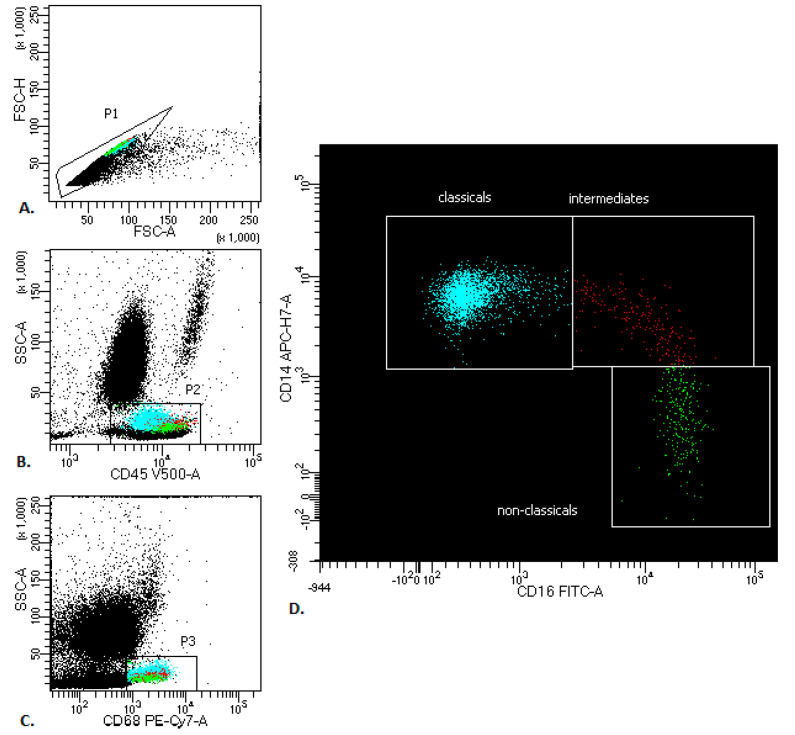
Representative monocyte gating strategy in peripheral blood of patients with lung cancer. (**A**) FSC-A vs. FSC-H plot: Gating the cells that have an equal area and height, thus removing clumps (greater FSC-A relative to FSC-H and debris (very low FSC). (**B**) CD45 vs. SSC-A plot: Broad selection of monocytes based on their SSC/CD45 properties. (**C**) CD68 vs. SSC-A plot: Broad selection of monocytes based on their SSC/CD68 properties. (**D**) CD16 vs. CD14 plot to gate the monocyte subsets: classicals (blue), intermediates (red) and non-classicals (green).

The median proportion of all PB monocytes in lung cancer patients was 7.1% (Q range: 5.9–8.6), vs. healthy donors: 6.4 (Q range: 4.4–7.5) There are three types of monocytes in PB: the classical monocytes with high expression of the CD14 cell surface receptor and no CD16 expression (CD14++CD16-), the non-classical monocytes with low/negative level of CD14 expression and co-expression of the CD16 receptor (CD14-/+CD16++) and the intermediate monocytes with expression of CD14 and expression of CD16 (CD14+CD16+) (Figure 1).

**Figure 2:**
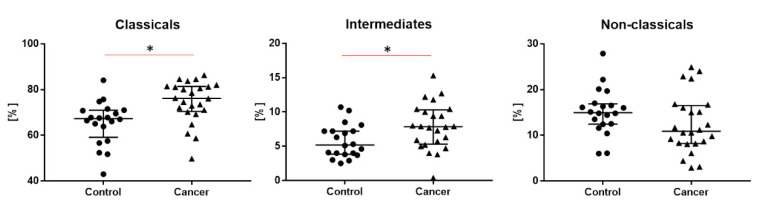
The differences of tree types of monocytes: classical, intermediate and non-classical monocytes between study and control groups. Graphs show the median values and quartile Q1–Q3, * *p* < 0.05.

We observed the highest proportion of the classical monocytes, a lower proportion of the non-classical monocytes and the lowest proportion of intermediate monocytes in both groups. We noticed a significantly higher proportion of classical monocytes (76.2 vs. 67.3, *p* < 0.05) and a higher proportion of intermediate monocytes in the lung cancer group than the in control group (7.9 vs. 5.2, *p* < 0.05) (Table 2 and Figure 2).

The median proportion of macrophages was higher in the clBALF then the hlBALF (33.4 vs. 22.4%, *p* < 0.05). In supplementary material there are shown plots from flow cytometry analysis with gating strategies for macrophages (Figure S1).

We analyzed the median proportion of cells with expression and geometric mean fluorescence (GMF) intensity of: CD62L, CD11c, CD18 and HLA-DR markers on three types of monocytes: classical, intermediate and non-classical in the study and control groups.

We compared the differences in expression of these antigens between monocyte groups: classical, intermediate and non-classical, both in the study group (Table 3) and in the control group (Table 4). In supplementary material there are shown plots with gating strategies and expression histograms from flow cytometry analysis (Figures S2–S5).

We noticed a higher GMF of CD62L on classical monocytes compared to intermediate monocytes and non- classical monocytes (respectively, 4425.5 vs. 883.5 vs. 556.5, *p* < 0.05) in the study group. In the study group, the median proportion of CD62L+ was higher on classical monocytes compared to intermediates and non-classical monocytes (95.0 vs. 35.4 vs. 16.2, *p* < 0.05).

For CD11c we observed higher GMF on intermediate monocytes, lower on classical and non-classical monocytes (respectively, 10,623 vs. 4712 vs. 6541, *p* < 0.05) in the study group. When we analyzed the median proportion of CD11+ cells we did not observe any differences.

For CD18 we also observed higher GMF on intermediate monocytes, lower on classical and non-classical monocytes (respectively, 2497.5 vs. 1923 vs. 1440, *p* < 0.05). When we analyzed the median proportion of CD18+ cells we observed the lowest proportion on non-classical monocytes compared to classical and intermediates.

**Figure 3:**
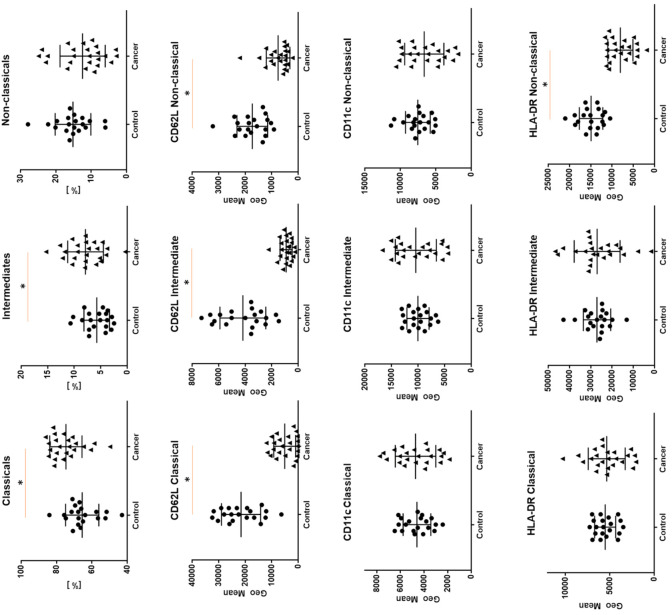
The differences between cancer group and study group for geometric means expression of CD62L, CD11c and HLA-DR on classical, intermediate, and non-classical monocytes. Graphs show the median values and quartile Q1–Q3 * *p* < 0.05.

For HLA-DR expression we also observed higher GMF for intermediate monocytes, a lower proportion for classical and non-classical monocytes (respectively, 29,320 vs. 5314 vs. 8654.5, *p* < 0.05). When we analyzed the median proportion of HLA-DR+ classical, intermediate, and non-classical cells we did not observe any differences. In addition to the exact characteristics of each group separately (Table 3 and Table 4), we compared the differences in the expression of the tested antigens on monocyte subsets between the study and control group. The differences of GMF between groups are presented in Figure 3 (for CD18 no differences were found, data not shown).

**Figure 4:**
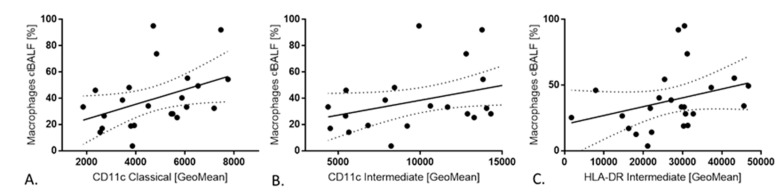
The correlations between monocytes in peripheral blood PB and macrophages in bronchoalveolar lavage fluid from cancer affected lung(clBALF). (**A**) Positive correlation between CD11c expression on classical monocytes in PB and macrophages in clBALF. (**B**) Positive correlation between CD11c expression on intermediate monocytes in PB and macrophages in clBALF. (**C**) Positive correlation between HLA-DR expression on intermediate monocytes in PB and macrophages in clBALF.

There were positive correlations between CD11c expression on classical monocytes with macrophages in clBALF (R = 0.43, *p* < 0.05) and CD11c expression on intermediates monocytes with macrophages in clBALF (R = 0.43, *p* < 0.05). We also observed a positive correlation between HLA-DR expression on intermediates monocytes and macrophages in clBALF (R = 0.45, *p* < 0.05) (Figure 4). We did not observe any correlation between any subsets of monocytes with macrophages from hlBALF.

## 3. Discussion

In this study we focused on monocytes and macrophages in NSCLC patients. We found a slightly higher number of monocytes in the blood of cancer patients than in healthy controls, which is in agreement with the results of Yang Hai et al. [21] where they showed the value of monocyte evaluation in lung cancer patient prognosis. Moreover, the results of our study present important differences in monocyte populations between patients and healthy donors.

We confirmed that there are three monocyte subpopulations: classical, intermediate and non-classical in fresh blood from NSCLC patients. We demonstrated that the most numerous was the population of monocytes with a classical phenotype. It is accepted that classical monocytes (CD14++CD16-) leave bone marrow and differentiate into intermediate monocytes (CD14+CD16+), and sequentially to non-classical (CD14-/+CD16++) monocytes in peripheral blood [22]. Classical monocytes were found to be primed for phagocytosis, innate immune responses and migration, but to have low proinflamatory potential [23]. Intermediate monocytes were the only subset expressing CC-chemokine receptor (CCR)5 and were well-suited for antigen presentation, cytokine secretion, apoptosis regulation, and differentiation. Non-classical monocytes are involved in complement and Fc gamma-mediated phagocytosis and their main function is adhesion [24,25]. Human classical monocytes leave the bone marrow in a CC-chemokine receptor 2 (CCR2)-dependent manner. In the steady state, classical monocytes can differentiate into intermediate monocytes, then differentiate into patrolling non-classical monocytes in circulation and participate in cancer surveillance by preventing tumor metastasis to lung [26]. During inflammation, classical and intermediate monocytes are tethered and invade tissue. Non-classical monocytes patrol the vessel wall and invade by the interaction of complementary pair of CX3CR1/CCL3 in a LAF/ICAM1-dependent manner [7]. It is now widely accepted that classical monocytes have the ability to differentiate into monocyte derived macrophages and dendritic cells [27] and play an integral part in shaping inflammation and its resolution in tissues. Intermediate monocytes express the highest levels of antigen presentation-related molecules [24,28]. Some studies shown that intermediate monocyte numbers are expanded in the blood of patients with systemic infections, implying that they must play an important role in the rapid defense against pathogens [29,30]. However, their exact role in immunity remains elusive as another study found that they are the main producers of IL-10 [31]. Whether these cells can produce pro- and anti-inflammatory mediators simultaneously or whether there are different kinetics of expression for these factors requires further exploration. In our study we observed a statistically significant higher percentage of classical and intermediate monocytes in the blood of patients with lung cancer compared to the control group. Rivas-Fuentes S. et al. [23] also analyzed the amount of these monocytes subsets with HLA-DR expression in NSCLS patients, but before and after chemotherapy, comparing to a control group. In our study, patients were prior to receiving any therapy. Opposite to our results, they found that HLA-DR+ classical and intermediate monocytes were decreased in patients before chemotherapy, compared to controls. After chemotherapy, the relative percentage of those subpopulations was restored. The percentage of classical and intermediate monocytes increased, approaching the values found in individuals without neoplasia, whereas non-classical (pro-inflammatory) monocytes were not modified. As in our work, non-classical monocytes did not differ between the study group and the control group. Our results and the observations of other researchers indicate that these two subpopulations: classical and intermediates monocytes may play a major role in patients with ongoing cancer. However considering the discrepancy in observations, it seems interesting to evaluate not only the percentages but also additional markers on these subpopulations.

In our study we determined not only the number of monocyte subtypes: classical, intermediate and non-classical but also analyzed the expression of surfaces markers: CD62L, CD11c, CD18, HLA-DR on these three monocyte subpopulations and we found differences between NSCLC patients and healthy donors.

We observed differences between monocyte subpopulations in the expression of test antigens, both in the test and control groups.

Expression of CD62L, CD11c, CD18 and HLA-DR was different on classical, intermediate or non-classical monocytes. The analyzed data were presented as positive cell percentage and the GMF intensity of CD62L, CD18, CD11c and HLA-DR. GMF was used to define and describe the mean intensity and level of antibody expression on the cell. In our study the differences were particularly emphasized when GMF of each of the antigens was taken into account in each group separately (Table 3—cancer patients, Table 4—healthy donors). Thus, in our opinion, the GMF value is more useful in the expression analysis of the tested antigens and more accurately reflects the differences in the expression of the studied markers between three monocyte subpopulations than assessing the percentage of cells expressing any particular antigen.

We observed that intermediate monocytes were characterized by the higher expression of CD11c, CD18 and HLA-DR than classical and non-classical monocytes in the study group. The expression of CD62L antigen was significantly higher on classical monocytes compared to intermediate and non-classical monocytes in the study group. Similar trends were observed in the control group (Table 4). Other researchers presented similar results of monocyte phenotyping. Abeles RD et al. [32] have shown that HLA-DR is an important marker that is highly expressed on intermediate monocytes and less so on non-classical monocytes. Mukherjee R et al. [33] have also shown that intermediate monocytes have a higher expression of HLA-DR and additionally have pro-angiogenic functions and immunosuppressive functions by production of IL-10. Thomas GD et al. [34] presented that HLA-DR and CD11c were higher on intermediate monocytes and together with CD14, CD16, CCR2 and CD36 may be the most informative markers that discriminate among the three monocyte populations. They presented that using these additional markers as filters, their revised gating scheme increases the purity of both intermediate and non-classical monocyte subsets to 98.8% and 99.1%, respectively.

Our results were similar to Thomas GD et al.’s [34] observations. They also obtained excellent results using CD62L as a marker that was more abundant on the classical subset of monocytes. Summing up the above data, we confirmed the importance of using additional markers to better select an individual monocyte group. A thorough analysis of GMF of the studied antigens on three monocyte subpopulations has enabled not only the detailed characteristics of each group individually, but also a comparison of these expressions between the healthy and the cancer groups. We presented for the first time assessed CD62L expression on three different monocyte subpopulations comparing the group of NSCLC patients with the healthy group. We observed higher CD62L antigen expression on all three monocyte subtypes in healthy donors than in the study group. It is known that in the state of physiology and proper functioning, monocytes present L-selectin CD62L on their surface. The high expression of CD62L implies a possible role of migration to lymph nodes and tissue and differentiation into a variety of antigen presenting cells: macrophages and dendritic cells [35]. In our study there was a high proportion of monocytes with low expression of CD62L in NSCLC patients. It reflects impaired adhesion and migration of monocytes and some weakness of immune response in cancer.

The CD11c antigen was another examined marker. Expression of CD11c on three different monocyte subpopulations did not differ between the study group and the control group. Almatroodi SA et al. [36] also did not observe differences in expression of CD11c in patients with NSCLC compared to non-cancer subjects. These results are consistent also with Mariotta S et al.’s [6] study outcomes, which suggested that NSCLC does not affect monocyte adherence and phagocytosis in lung cancer patients compared to healthy controls.

In the case of HLA-DR expression, we noticed higher expression on non-classical monocytes in the control group than in study group, while classical and intermediate monocytes with HLA-DR expression did not differ between the study and control groups. Thus, it seems interesting to analyze HLA-DR antigen expression, not only on classical and intermediate subpopulations, but also on non-classical (CD14-/+CD16++). We focused on these subsets for the first time.

Some other studies have reported reduced HLA-DR expression on blood monocytes in human cancer, but mainly on CD14 positive monocytes. Vuk-Pavlović S et al. [4] presented CD14(+) monocytes exhibiting reduced expression of HLA-DR molecules in prostate cancer patients and suppressed immune cell function by these cells in vitro. CD14+ HLA-DRNeg/Low peripheral monocytes have immunosuppressive functions in patients with different types of cancer and increase was connected with poor prognosis in cancer [37,38]. Huang A et al. [39] have shown that both frequency and absolute number of CD14(+)HLA-DR(-/low) cells were significantly increased in the peripheral blood of NSCLC patients compared with that of the healthy subjects and indicated an association with metastasis, poor response to chemotherapy and progression-free survival. Tian T et al. [40] also focus on CD14(+)HLA-DR-/low cells and indicated that increased circulating CD14(+)HLA-DR-/low myeloid-derived suppressor cells (MDSCs) are associated with poor prognosis in patients with small-cell lung cancer (SCLC). These results indicate the role of reduced HLA-DR expression on monocytes in lung cancer patients, which may mediate tumor immunosuppression and might thus represent a potential target for therapeutic intervention.

In our study we found positive correlations between CD11c expression on classical monocytes and CD11c expression on intermediate monocytes with macrophages from clBALF and positive correlation between HLA-DR expression on intermediates monocytes and macrophages from clBALF. In contrast, we did not observe any correlation between any subsets of monocytes with macrophages in the BALF from the “healthy lung”. Some studies have presented that surface markers such as F4/80, MHCII, and CD11c are up-regulated once monocytes enter tissue, while Ly6C and CD11b are down-regulated, but these markers are often expressed along a continuum between blood monocytes and cells in tissue [41]. Our results could indicate that monocytes with increased CD11c expression are more likely to enter tissues and become macrophages. It is also not clear if monocytes can directly support angiogenesis, or first require differentiation and acquisition of macrophage-associated functions [42]. The plasticity of macrophages with an outcome of tumor suppression or tumor growth highlights the challenges in targeting these cells in cancer therapies (our results, in press). Defining human monocyte subsets which may correlate with macrophages in the tumor microenvironment, as well as antigen expression assessment would present opportunity for rapid clinical therapies.

Our results and the above-presented data of other studies imply that not only the intermediate and classical, but also non-classical monocytes may play an important role in lung cancer patients. Understanding the changes in monocyte phenotype with the expression of specific markers, mainly HLA-DR and CD11c in NSCLC may allow the development of novel biomarkers or therapeutic strategies.

## 4. Materials and Methods

### 4.1. Patients

PB and BALF were obtained from 38 patients undergoing diagnostic bronchoscopy for investigation of lung tumors recruited through the Department of Surgery, National Institute of Tuberculosis and Lung Diseases, Warsaw, Poland. Each patient had provided written informed consent (the Military Institute of Medicine Ethics Committee, 47/WIM/2017) before each diagnostic procedure. Primary NSCLC confirmed by histological examination constituted an inclusion criterion. The exclusion criteria were as follows: any type of prior or recent anti-cancer therapy, clinical signs of infection, chronic obstructive pulmonary disease (COPD), autoimmune diseases, immunosuppressive treatment. Ten patients without confirmation of NSCLC and four patients with diagnosis of metastatic adenocarcinoma were also excluded from the study group. Further exclusion criteria were established following BALF macro-scale and microscopic examination.

Finally, the study group consisted of 24 patients with confirmed primary NSCLC. There were 15 women and 9 men; mean age: 69.3 ± 6.4 years; range (min–max): 53–83 years. The control group consisted of 20 healthy donors—from them only PB samples were collected. Demographic details of the participants and clinical details of lung cancer patients were presented in Table 1. NSCLC staging was applied in this study using the eighth TNM classification of malignant tumors [43].

### 4.2. Material

Five milliliters of blood was collected from each patient. Bronchoalveolar lavage was performed during a routine bronchofiberoscopy in the course of lung cancer diagnosis from the same patient and on the same day after blood collection. BALF was taken from the cancerous lung (clBALF) and from the healthy lung (hlBALF) of the same patient during the same procedure. An amount 100 mL of 0.9% NaCl solution was instilled to each lung and immediately removed. BALF processing was realized according to the recommendations in [44].

### 4.3. Flow Cytometry Analysis

Monocyte subtyping and macrophage counts were performed by flow the cytometry method with panel of monoclonal antibodies using FACS Canto II BD flow cytometry (Becton Dickinson, Franklin Lakes, NJ, USA). For surface marker detection, cells were stained with fluorescently labeled antibodies: CD16- FITC, CD62L- PE, CD11c-PerCP-Cy5.5, CD18- APC, CD14- APC-H7, HLA-DR- V450, CD45-V500 (BD Biosciences) for 20 min at room temperature. For intracellular CD68-PE-Cy7 detection (macrophages identification) (BD Biosciences) the additional step with IntraStain (Dako, Glostrup, Denmark) for fixation and membrane permeabilization was carried out. After washing, cells were analyzed within 2 h. For each sample, a minimum of 100,000 events were collected. Data were analyzed with DIVA Analysis software 8.0.1 (BD) and Infinicyt 1.8 Flow Cytometry (Cytognos, Salamanca, Spain).

### 4.4. Statistical Analysis

The Statistica 13.0 software (TIBCO Software, Palo Alto, CA, USA) was used for statistical analysis. The results are expressed as medians with interquartile range (Q1–Q3) and median of GMF of markers on monocytes. For group comparison the Mann–Whitney test and Kruskal–Wallis tests were used. Relations between the quantitative variables were analyzed by Spearman correlations. A *p* < 0.05 was considered as statistically significant.

## 5. Conclusions

In this study we confirmed the presence of various monocyte subtypes in the blood with predominance of classic monocytes CD14++CD16- and a higher proportion of classical and intermediate monocytes in NSCLC patients than in healthy subjects. Our observation that intermediate monocytes (CD14+CD16+) with CD11c+ and HLA-DR+ expression correlation with the amount of macrophages from the lung cancer microenvironment may indicate role a of these cells in cancer immunity. A high proportion of monocytes with low expression of CD62L indicates participation of monocytes in attenuation of anticancer response. The detection and monitoring of the presented monocyte subsets in the blood might be a useful test in lung cancer.

## Figures and Tables

**Table 1:** Demographic details of lung cancer patients and control subjects.

Groups	*n*	Age (yr) Mean ± SD	Sex M/F	Stage I/II/III/IV	Subtypes AD/SQCC/LCC/AS
**Cancer**	24	69.3 ± 6.4	9/15	15/6/3/0	16/5/2/1
**Excluded from the study:**					
- no cancer cells	10	71.4 ± 8.3	6/4	-	-
- metastatic cells	4	60.5 ± 10.8	1/3	-	-
**Control group**	20	51.9 ± 9.8	1/10	-	-

Abbreviations: *n*: Number; SD: Standard deviation; M: Male; F: Female; AD: Lung adenocarcinoma; SQCC: Squamous cell lung carcinoma; LCC: Large cell neuroendocrine carcinoma; AS: adenosquamous cell lung carcinoma.

**Table 2:** Median proportion of tree types of monocytes in study and control groups and BALF macrophages in study group. (* *p* < 0.05 Mann–Whitney U test).

Cells	Study Group PB % Median (Q1–Q3)	Control Group PB % Median (Q1–Q3)	Study Group cBALF % Median (Q1–Q3)	Study Group hBALF % Median (Q1–Q3)
**All Monocytes**	7.1 (5.9–8.6)	6.4 (4.4–7.5)	-	-
**Classical monocytes** * CD14++CD16-	76.2 (70.7–81.3) *	67.3 (60.7–70.9) *	-	-
**Intermediate monocytes** * CD14+CD16+	7.9 (5.5–10.2) *	5.2 (3.9–0.2) *	-	-
**Non classical monocytes** CD14-/+CD16++	10.9 (8.3–16.4)	14.9 (12.5–16.8)	-	-
**Macrophages** * CD68++	-	-	33.4 (22.3–48.8) *	22.4 (11.5–30.9) *

Abbreviations: cBALF: bronchoalveolar lavage fluid from cancer affected lung; hlBALF: bronchoalveolar lavage fluid from healthy symmetrical lung.

**Table 3:** Median proportion and median geometric means expression of CD62L, CD11c, CD18 and HLA-DR markers on three types of monocytes in study group. * *p* < 0.05 as statistically significant.

Expression of:	Classical Monocytes a Geometric Mean (Median (Q1–Q3)) % (Median (Q1–Q3))	Intermediate Monocytes b Geometric Mean (Median (Q1–Q3)) % (Median (Q1–Q3))	Non-classical Monocytes c Geometric Mean (Median (Q1–Q3)) % (Median (Q1–Q3))	Kruskal–Wallis Test *p* < 0.05 *
CD62L	**4425.5 (879.5–8793.5)**	**883.5 (509–1220)**	**556.5 (494.5–983.5)**	0.0007 *a–b, a–c
	95.0 (80.2–98.6)	43.4 (26–65.7)	26.7 (14.1–50.2)	0.0001 *a–b, a–c
CD11c	**4712 (3460–6069)**	**10623 (6820–13828)**	**6541 (4637-8732)**	0.0000 *a–b, b–c
	98 (96–99)	100 (99.1–100)	95.1 (87.9–98.1)	-
CD18	**1923 (1572.5–2407.5)**	**2497.5 (1926.5–2993.5)**	**1440 (1178–1831.5)**	0.0000 *a–c, b–c
	80.6 (48.7–94.3)	96.9 (84.9–99.5)	44.9 (34.7–62)	0.0000 *a–b, b–c
HLA-DR	**5314 (3977.5–6540)**	**29320 (21456–31255)**	**8654.5 (5483.5–10761.5)**	0.0000 *a–b, b–c
	96.4 (97.5–99.6)	99.8 (97.9–100)	90.6 (83–98)	-

**Table 4:** Median proportion and median geometric means expression of CD62L, CD11c, CD18 and HLA-DR markers on three types of monocytes in control group. * *p* < 0.05 as statistically significant.

Expression of:	Classical Monocytes a Geometric Mean (Median (Q1–Q3)) % (Median (Q1–Q3))	Intermediate Monocytes b Geometric Mean (Median (Q1–Q3)) % (Median (Q1–Q3))	Non classical Monocytes c Geometric Mean (Median (Q1–Q3)) % (Median (Q1–Q3))	Kruskal–Wallis Test *p* < 0.05 *
CD62L	**22850.5 (15397.5–27104)**	**3556. 5 (2983–5670)**	**1727 (1237.5–2104.5)**	0.0000 * a–b–c
	95.6 (90.9–97.4)	35.4 (28.3–50.9)	16.2 (10.4–24.0)	0.0001 * a–b–c
CD11c	**4536 (3727–5703)**	**9954 (8576–11728.5)**	**7396.5 (6297.5–8275)**	0.0000 * a–b–c
	82.9 (70.9–92.9)	88 (82.1–97.6)	85.6 (76.9–89.1)	-
CD18	**1730 (1460–2350.5)**	**2284 (1899–2891)**	**1499 (1210–1922)**	0.0000 *a–c, b–c
	81.4 (50.1–96.1)	95.8 (85.5–99.1)	41.9 (37.1–69.2)	0.0000 *a–b, b–c
HLA–DR	**5777.5 (4495–6582)**	**25483.5 (24065–30712.5)**	**14999.5 (12732.5–16474)**	0.0000 * a–b–c
	69.3 (57.2–77.1)	93.3 (85.1–96.45)	77.8 (70.6–84.6)	0.0001 * a–b, b–c

## Supplementary Materials

The following are available online at https://www.mdpi.com/2072-6694/12/9/2513/s1, Figure S1: Flow cytometry analysis of alveolar macrophage (AMs) in hlBALF (A) and clBALF (B), Figure S2: The gating strategy of monocytes and CD11c expression histograms with statistic in PB from representative cancer patient, Figure S3: The gating strategy of monocytes and CD18 expression histograms with statistic in PB from representative cancer patient, Figure S4: The gating strategy of monocytes and HLA-DR expression histograms with statistic in PB from representative cancer patient, Figure S5: The gating strategy of monocytes and CD62L expression histograms with statistic in PB from representative cancer patient.
