# Supplementary material for: Blood Monocyte Subsets with Activation Markers in Relation with Macrophages in Non-Small Cell Lung Cancer
Source: Cancers (Basel). 2020 Sep 4;12(9):2513. doi: 10.3390/cancers12092513 (PMC7563629; doi:10.3390/cancers12092513)
Supplement: Supplementary file 1 [file cancers-12-02513-s001.pdf]

Supplementary Materials

Blood Monocyte Subsets with Activation Markers in Relation with Macrophages in Non-Small Cell Lung Cancer

Iwona Kwiecień, Elżbieta Rutkowska, Małgorzata Polubiec-Kownacka, Agata Raniszewska, Piotr Rzepecki and Joanna Domagała-Kulawik

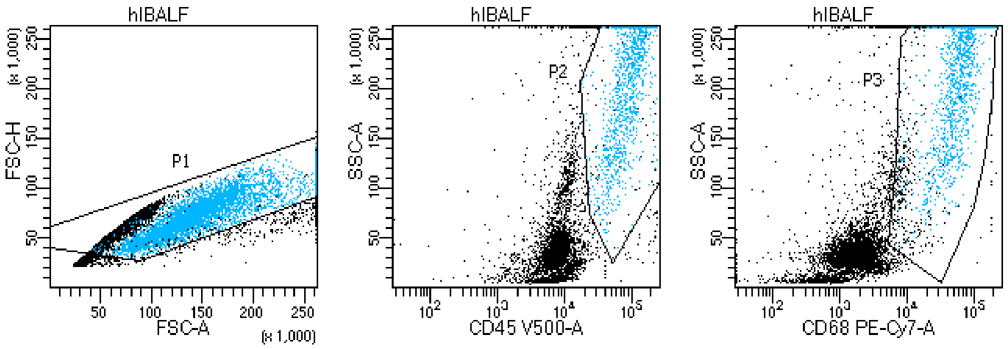

Tube: Macrophages hBALF

Population	#Events	%Parent	%Total
All Events	10,000	####	100.0
P1	9,017	90.2	90.2
P2	4,328	48.0	43.3
P3	4,289	99.1	42.9
Macrophages	4,289	42.9	42.9

A

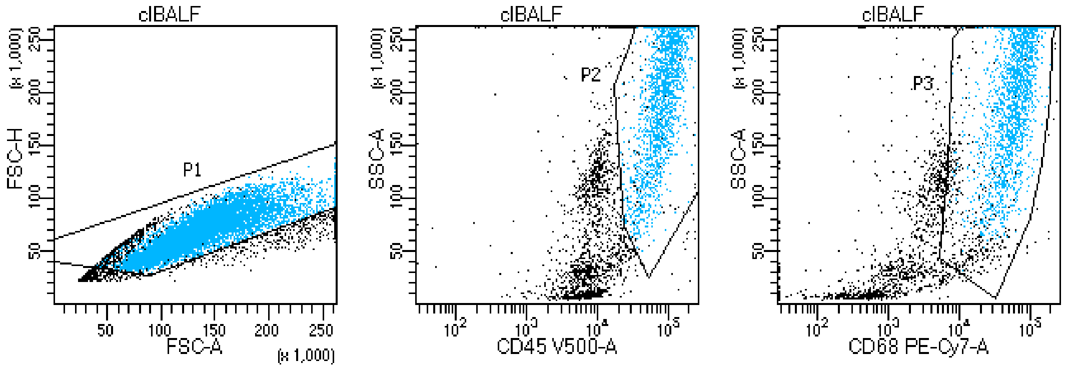

Tube: Macrophages cBALF

Population	#Events	%Parent	%Total
All Events	10,000	####	100.0
P1	8,994	89.9	89.9
P2	7,877	87.6	78.8
P3	7,845	99.6	78.4
Macrophages	7,845	78.4	78.4

B

Figure S1. Flow cytometry analysis of alveolar macrophage (AMs) in hlBALF (**A**) and clBALF (**B**). AMs were gated as CD45+bright/CD68+bright without clumps (greater FSC-A relative to FSC-H) and debris (very low FSC).

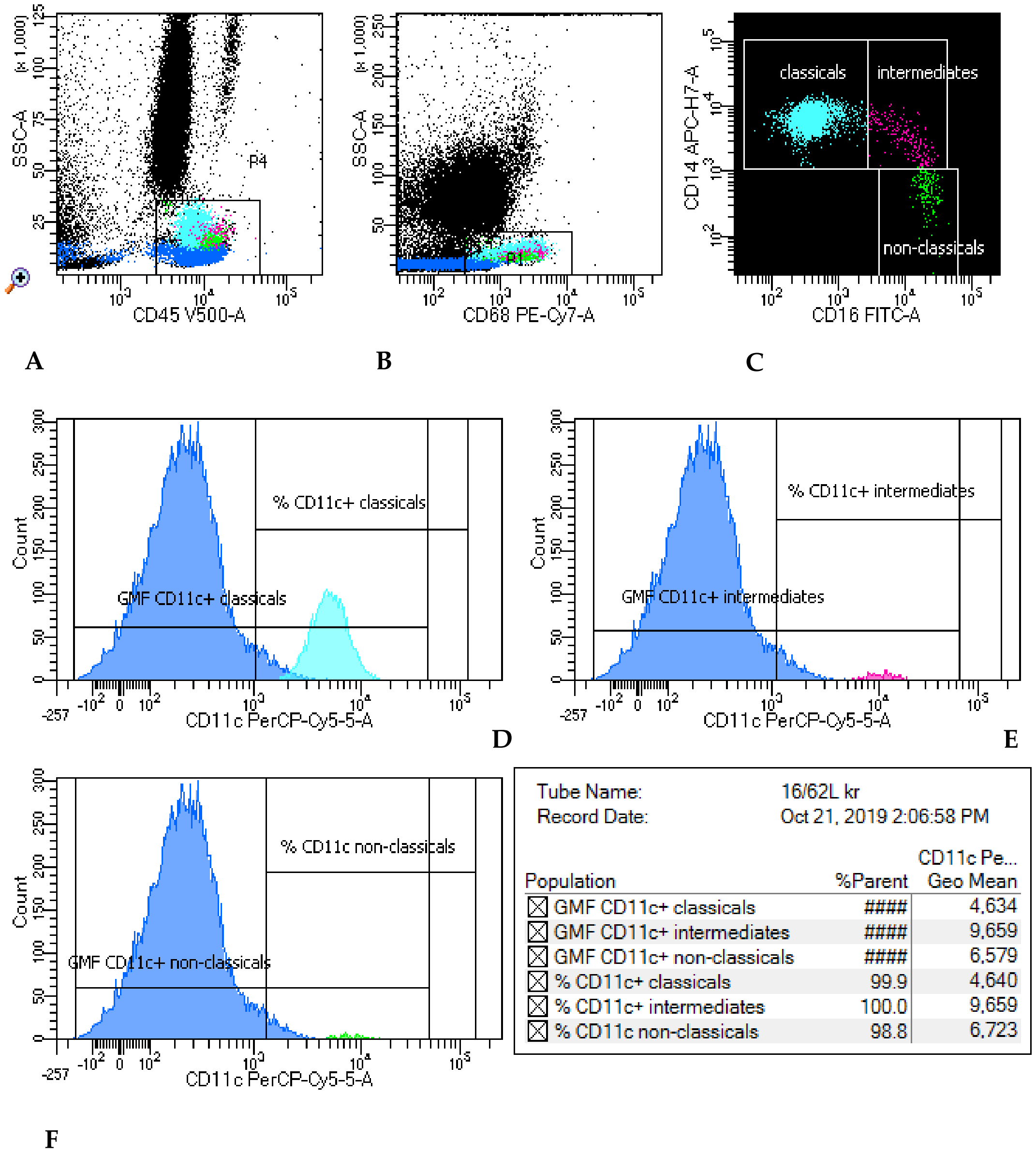

Population	%Parent	CD11c Pe... Geo Mean
GMF CD11c+ classicals	####	4,634
GMF CD11c+ intermediates	####	9,659
GMF CD11c+ non-classicals	####	6,579
% CD11c+ classicals	99.9	4,640
% CD11c+ intermediates	100.0	9,659
% CD11c non-classicals	98.8	6,723

Figure S2. The gating strategy of monocytes and CD11c expression histograms with statistic in PB from representative cancer patient. Cells with fluorescence on histograms were visualized with 'logicle' displays. (**A**) CD45 vs. SSC-A plot: Broad selection of monocytes based on their SSC/CD45 properties. (**B**) CD68 vs. SSC-A plot: Broad selection of monocytes based on their SSC/CD68 properties. (**C**) CD16 vs. CD14 plot to gate the monocyte subsets: classicals (blue), intermediates (red) and non-classicals (green). (**D**) Histogram with CD11c expression on classical monocytes (with percentages values and GMF). (**E**) Histogram with CD11c expression on intermediate monocytes. (**F**) Histogram with CD11c expression on non- classical monocytes.

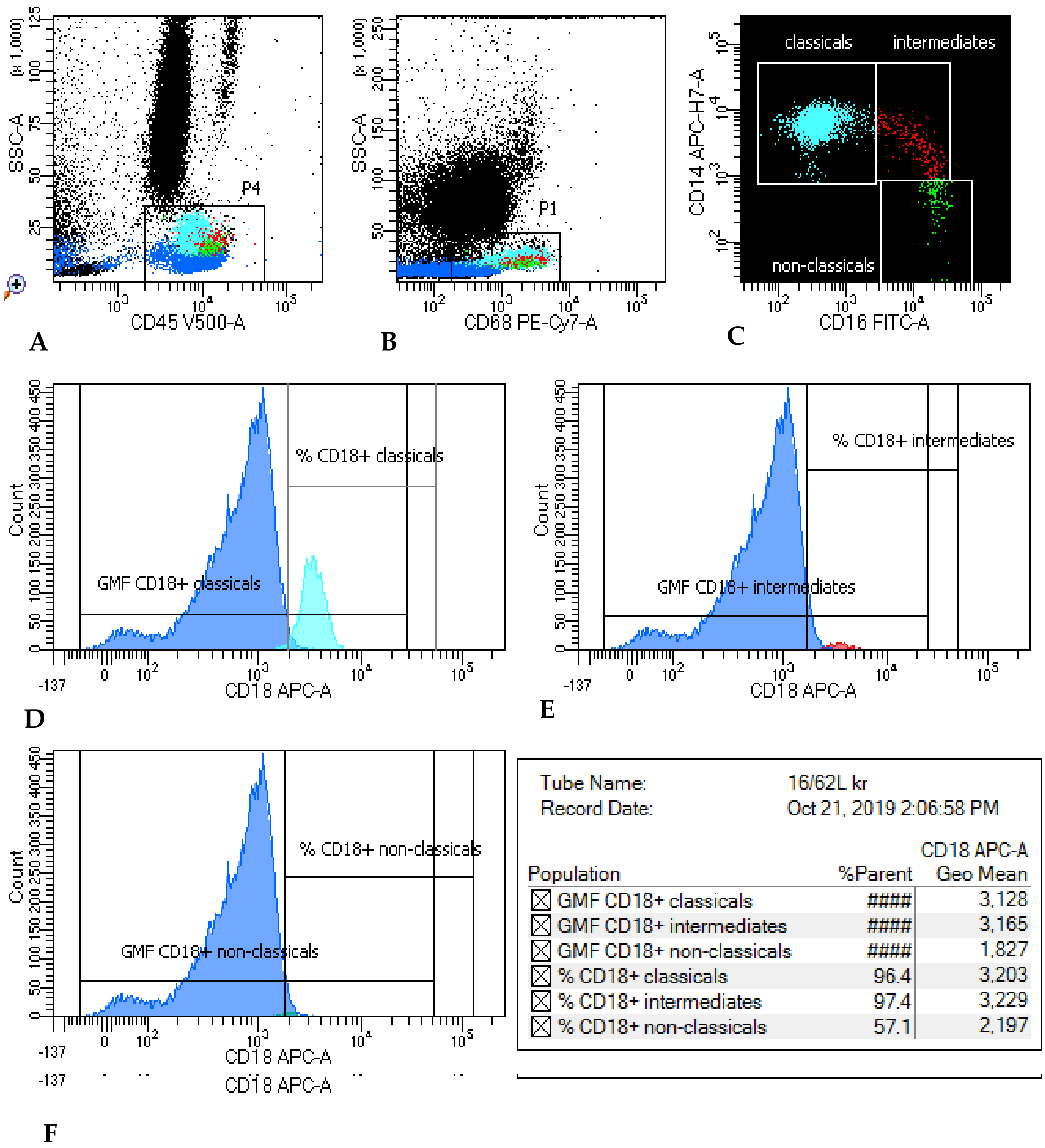

Figure S3. The gating strategy of monocytes and CD18 expression histograms with statistic in PB from representative cancer patient. Cells with fluorescence on histograms were visualized with 'logicle' displays. (**A**) CD45 vs. SSC-A plot: Broad selection of monocytes based on their SSC/CD45 properties. (**B**) CD68 vs. SSC-A plot: Broad selection of monocytes based on their SSC/CD68 properties. (**C**) CD16 vs. CD14 plot to gate the monocyte subsets: classicals (blue), intermediates (red) and non-classicals (green). (**D**) Histogram with CD18 expression on classical monocytes (with percentages values and GMF). (**E**) Histogram with CD18 expression on intermediate monocytes. (**F**) Histogram with CD18 expression on non- classical monocytes.

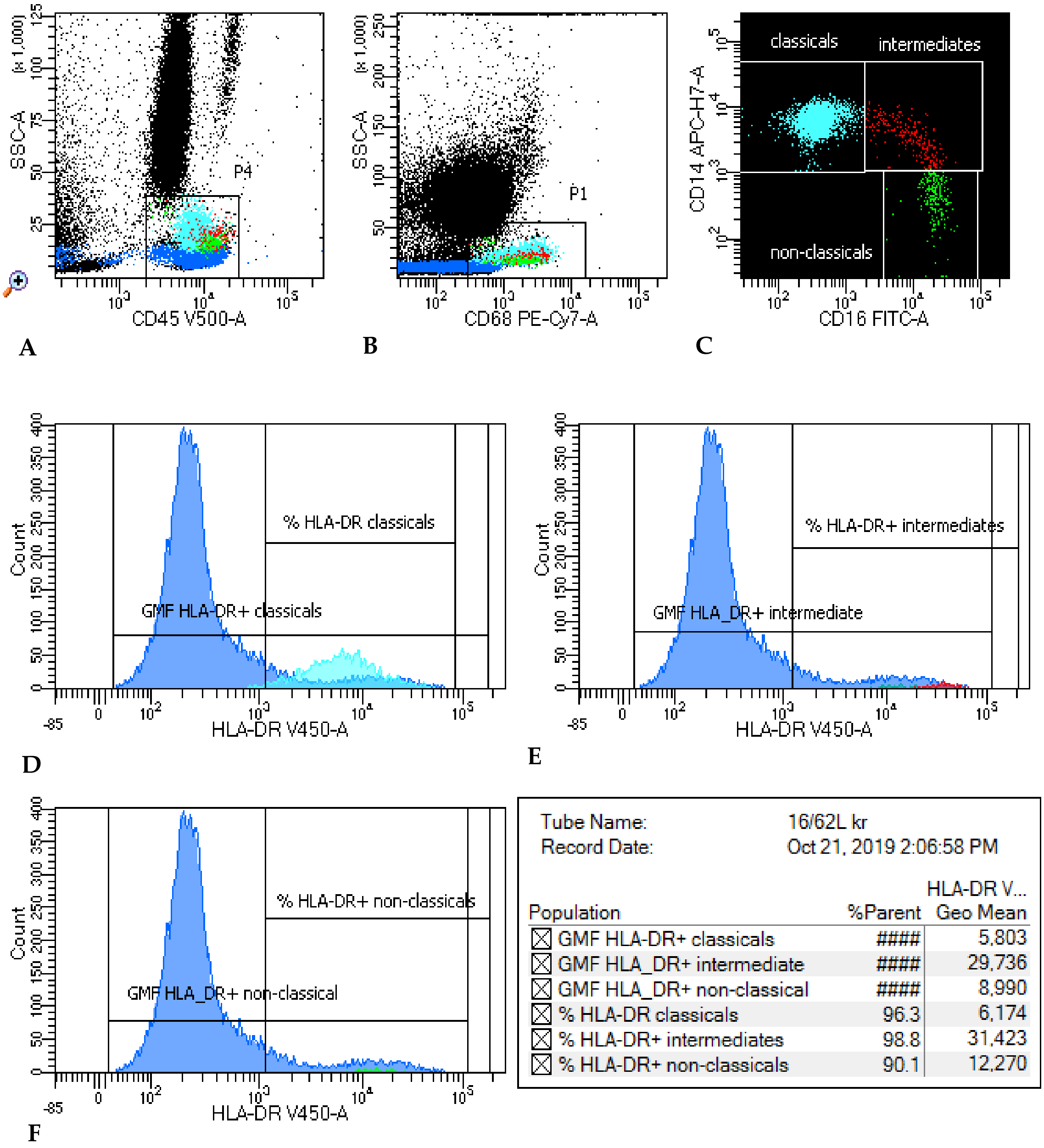

Figure S4. The gating strategy of monocytes and HLA-DR expression histograms with statistic in PB from representative cancer patient. Cells with fluorescence on histograms were visualized with 'logicle' displays. (**A**) CD45 vs. SSC-A plot: Broad selection of monocytes based on their SSC/CD45 properties. (**B**) CD68 vs. SSC-A plot: Broad selection of monocytes based on their SSC/CD68 properties. (**C**) CD16 vs. CD14 plot to gate the monocyte subsets: classicals (blue), intermediates (red) and non-classicals (green). (**D**) Histogram with HLA-DR expression on classical monocytes (with percentages values and GMF). (**E**) Histogram with HLA-DR expression on intermediate monocytes. (**F**) Histogram with HLA-DR expression on non- classical monocytes.

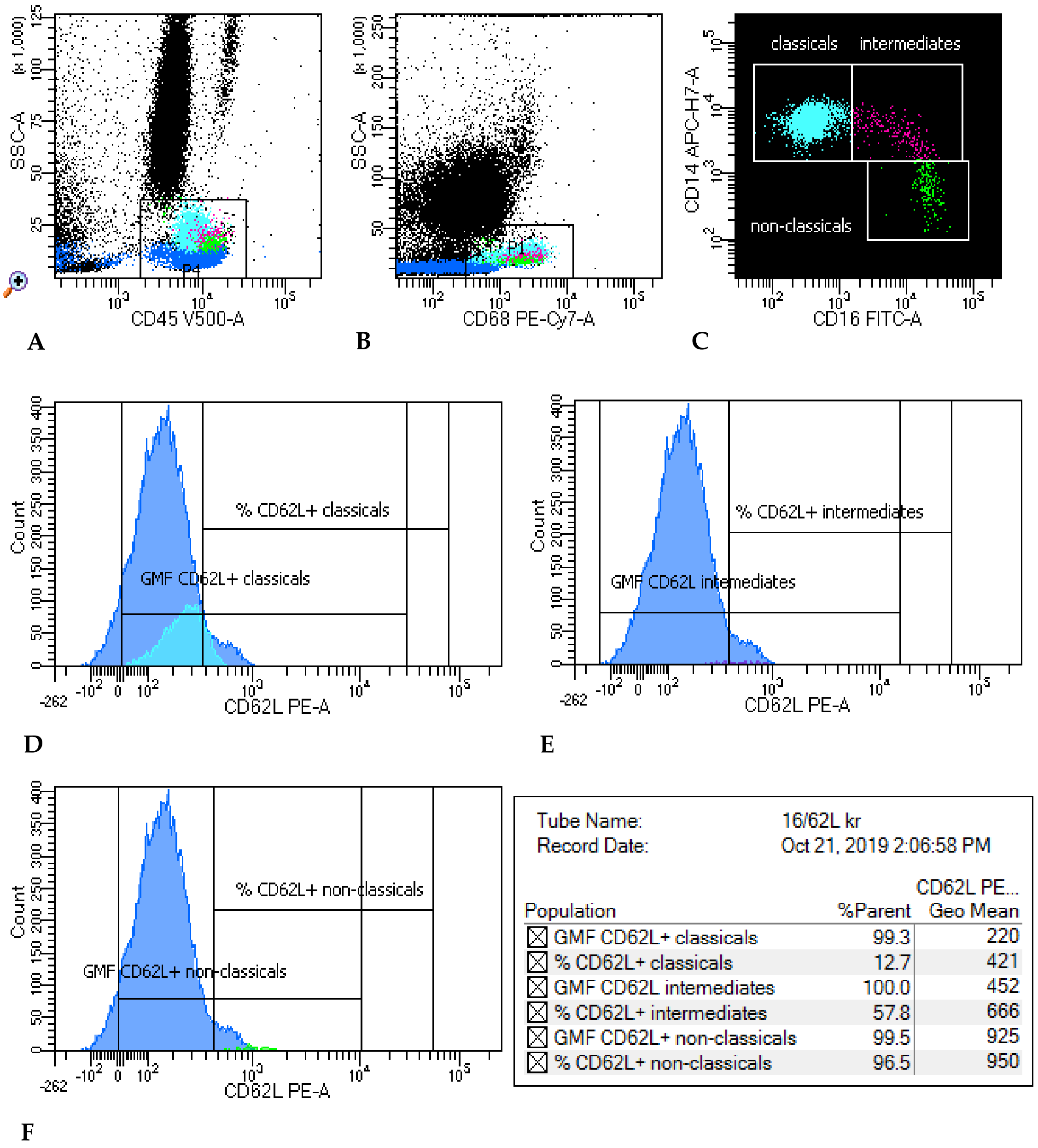

Population	%Parent	CD62L PE... Geo Mean
GMF CD62L+ classicals	99.3	220
% CD62L+ classicals	12.7	421
GMF CD62L intemediates	100.0	452
% CD62L+ intermediates	57.8	666
GMF CD62L+ non-classicals	99.5	925
% CD62L+ non-classicals	96.5	950

Figure S5. The gating strategy of monocytes and CD62L expression histograms with statistic in PB from representative cancer patient. Cells with fluorescence on histograms were visualized with 'logicle' displays. (**A**) CD45 vs. SSC-A plot: Broad selection of monocytes based on their SSC/CD45 properties. (**B**) CD68 vs. SSC-A plot: Broad selection of monocytes based on their SSC/CD68 properties. (**C**) CD16 vs. CD14 plot to gate the monocyte subsets: classicals (blue), intermediates (red) and non-classicals (green). (**D**) Histogram with CD62L expression on classical monocytes (with percentages values and GMF). (**E**) Histogram with CD62L expression on intermediate monocytes. (**F**) Histogram with CD62L expression on non- classical monocytes.

© 2020 by the authors. Licensee MDPI, Basel, Switzerland. This article is an open access article distributed under the terms and conditions of the Creative Commons Attribution (CC BY) license (http://creativecommons.org/licenses/by/4.0/).
